# Application of the JULES-crop model and agrometeorological indicators for forecasting off-season maize yield in Brazil

**DOI:** 10.1016/j.heliyon.2024.e29555

**Published:** 2024-04-11

**Authors:** Amauri Cassio Prudente Junior, Murilo S Vianna, Karina Willians, Marcelo V Galdos, Fabio R. Marin

**Affiliations:** a University of São Paulo, “Luiz de Queiroz” College of Agriculture, Piracicaba, SP, 13418-900, Brazil; b UK Met Office, Fitzroy Road, Exeter, EX1 3PB, UK; c Global Systems Institute, University of Exeter, Exeter, EX4 4PY, UK; d Institute of Physics- University of São Paulo, São Paulo-SP, 05508-090, Brazil; e Rothamsted Research, Harpenden, Hertfordshire, AL5 2JQ, UK

**Keywords:** *Zea mays*.L, Yield forecast, Large-scale analysis

## Abstract

*Zea mays* L is a crucial crop for Brazil, ranking second in terms of production and sixth in terms of exports. In Brazil, the second season, or off-season, accounts for 80 % of the overall maize output, which primarily occurs after the soybean main season. A maize yield forecast model for the off-season was developed and implemented throughout Brazilian territory due to its importance to the country's economy and food security. The model was built using multiple linear regressions that connected outputs simulated from a land surface model used in large-scale analysis for agriculture (JULES-crop), to agrometeorological indicators. The application of the developed model occurred every 10 days from the sowing until the maturation. A comparison of the forecasting model was verified with the official off-season maize yields for the years 2003–2016. Agrometeorological indicators during the reproductive phase accounted for 60 % of the interannual variability in maize production. When outputs simulated by JULES-crop were included, the forecasting model achieved Nash-Sutcliffe modeling efficiency (EF) of 0.77 in the maturation and EF = 0.72 in the filling-grain stage, suggesting that this approach can generate useful predictions for final maize yield beginning on the 80th day of the cycle. Outputs of JULES crop enhanced modeling performance during the vegetative stage, reducing the standard deviation error in prediction from 0.59 to 0.49 Mg ha^−1^.

## Highlights

• A yield-predicting approach was proposed based on JULES-crop outputs and agrometeorological indicators.
• The yield forecast model was tested at the national scale for off-season maize.
• The forecasting approach demonstrated high performance in predicting yield from the 80th day of the cycle.
• Maize yield inter-annual variability was explained by agrometeorological indicators in 60 %, during the reproductive stage.

## 1 INTRODUCTION

Maize, an annual crop, holds significant relevance in food security and the global economy due to its nutritional content [1]. Maize ranks as the second most cultivated crop in Brazil, producing 115.2 Tg in 2021 (BRASIL, 2022)[2], thereby securing its position as the third-largest producer in the world. Approximately 88 % of the Brazilian production of maize is designated for animal feed (CONAB, 2019; [3]), while the remainder is utilized for human consumption, mainly in the northwest semiarid [4] and for silage [5]. Environmental conditions in Brazil prompt two growth seasons for maize: the first season or main season (sown between September and December) and the second season or off-season maize (sown between January and April) following soybean cultivation [6,7]. Presently, maize in off-season constitutes 80 % of Brazil's overall production (CONAB, 2021), marking it as the country's most crucial growing season.

Brazilian maize plays a vital role in global food security and serves as a cornerstone in various supply chains, underscoring the necessity for accurate maize yield estimates in Brazil. Regional or national-scale yield forecasting can generate early warnings to institutions and stakeholders, enabling preemptive action before affecting production by severe climatic conditions [8]. Additionally, the maize supply chain significantly impacts sectors such as energy, animal nutrition, agriculture, and marketing, all of which stand to benefit from early yield forecasting. Anticipating yields could also facilitate adjustments in the importation of foods and agricultural markets regulation (Liu and Basso, 2019) while mitigating price volatility resulting from unforeseen speculative activities and yield losses [9].

Traditional yield forecasting systems have evolved from relying solely on crop scouting and on-farm surveys to incorporating crop management and weather data (Bannayan and Crout, 1999) [10]. Modern forecasting systems integrate agrometeorological indicators, crop modeling and remote sensing [[11], [12], [13]]. However, current studies have identified limitations in these systems, highlighting the need to reduce model parametrization errors and identify causal yield losses factors and data uncertainties at regional scales [14,15]. Given the Brazilian status as the third worldwide maize producer and the importance of yield forecasting in mitigating price volatility, there is a pressing need for advancements in maize production prediction strategies in Brazilian territory.

In various regions of Brazil, maize yield forecasts have been developed using diverse methods, including data from remote sensing [16], simulations in crop models integrated with agrometeorological indicators ([4,17]; Duarte et al., 2020) and edaphoclimatic indicators [18]. Even with an increase in the relevance of maize in the off-season for Brazilian agriculture in recent years, none of these studies have addressed off-season maize production on a national scale. Additionally, crop models utilized for yield forecasts in Brazil, such as FAO-AEZ, Aquacrop, and CERES-Maize have limitations in integrating biosphere-atmosphere processes with crop physiology, lacking the capability to incorporate CO2, water, and energy fluxes for simulating crop growth on a large scale. In efforts to enhance the representation of plant development and growth in Earth systems modeling, Osborne et al. (2015) introduced a crop parametrization to the Joint UK Land Environment Simulator (JULES) a land surface model [19,20], named as JULES-crop. JULES-crop demonstrates improved abilities to simulate maize under irrigated [21] and rainfed ([22]; Prudente Jr et al., 2022) conditions. However, despite its capabilities, it has not yet been utilized for yield forecasting at a national scale for off-season maize in Brazilian territory. Given the significance of Brazilian maize production in global food security, as the third-largest producer worldwide, and the pressing need to address the lack of predictive models on a national scale for off-season maize production as a strategy to mitigate impacts on pricing and grain storage in the face of impending climate change scenarios, this study introduces a yield forecasting approach for maize. The approach is based on agrometeorological indicators and utilizes the JULES-crop model, aiming to achieve the following objectives: (a) Recognize the factors explaining variability of maize yield across distinct phenological stages. (b) Analyze the yield prediction model by utilizing outputs of JULES-crop in water-limited and potential conditions in conjunction with agrometeorological indicators. (c) Evaluate the maize off-season yield prediction at a scale in national level for Brazilian territory.

## 2 Material and methods

### 2.1 JULES-crop model description

Crop development operates in JULES-crop through a development index (DVI) ranging from −2 to 2. Each value within this range corresponds to a specific stage in the crop's growth cycle.

• A DVI value of −2 indicates the period before sowing,
• -1 indicates the date of sown,
• 0 indicates emergence,
• 1 marks the reproductive stage start, and
• 2 signifies the end of the crop cycle, typically coinciding with harvest.

The DVI serves as a basis for modeling various aspects of crop growth, including carbon partitioning, and specific leaf area (SLA), during stages of development (vegetative, senescence, and harvest timing). It is calculated based on the effective temperature (Teff) accumulated during the cycle, also referred to as rising degree days ([21]; Osborne et al., 2015), as outlined in equation (1):

(1) $$Teff=\left\{\begin{array}{c} 0\;for\;T<Tb\\ T-Tb\;for\;Tb\leq T\leq To\\ \left(To-Tb\right)\left(1-\frac{T-To}{Tm-To}\right)\;for\;To<T<Tm\\ 0\;for\;T\geq Tm \end{array}\right\}$$

where To is the optimal temperature for crop growth and development; Tm is the maximum temperature, and Tb is the base temperature (i.e crop develops fastly when the temperature is near to the optimal temperature). Every maize temperature was determined using data from Birch et al. [23] and Williams et al. [21].

JULES estimates the DVI progress between the emergency toward flowering based on Loomis (1992) approach, in which the day length (*P*) is lower than (greater than) a specific crop critical photoperiod (P_*crit*_) for long days. The sensitivity degree to the photoperiod is indicated by the parameter P_*sens*_. equation (2) describes the relative photoperiod effect (RPE) as demonstrated by Connor and Loomis [24]:

(2) $$\text{RPE}=1-\left(P-P_{\text{crit}}\right)P_{\text{sens}}$$

The DVI rate increases is calculated as described in equation (3), where TT_emr_ is the thermal time between sown date and emergence, TT_veg_ is the thermal time between emergence and flowering and TT_rep_ is the thermal time between flowering and harvest:

(3) $$\frac{\text{dDVI}}{\text{dt}}=\left\{\begin{array}{c} \begin{array}{c} \frac{\text{Teff}}{\text{TTemr}}\;\text{for}\;-1\leq \text{DVI}<0\\ \left(\frac{\text{Teff}}{\text{TTveg}}\right)\text{RPE}\;\text{for}\;0\leq \text{DVI}<1\; \end{array}\\ \frac{\text{Teff}}{\text{TTrep}}\;\text{for}\;1\leq \text{DVI}<2\; \end{array}\right\}$$

JULES-crop divides net primary productivity (NPPacc) among a stem reserve pool and each plant structure for crop growth simulations. The carbon partition is determined by parameters specified by users. To obtain partitions factors of some crop for each carbon pool (pi), equation (4) was used:

(4) $$\text{pi}=\frac{\exp\left(\alpha i+\beta iDVI\right)}{\sum_{j}\exp\;\left(\alpha j+\beta jDVI\right)}$$

where j represents leaf, stem, harvest component (grain in maize), and root. αi and βi are adjusted numerical constants for observational data. $\sum_{j}pj=1$. The carbon partitioning to the stem undergoes a later adjustment of remobilization (between the reserve and the structure).

When DVI reaches the threshold, the initialization of carbon pools is triggered to the user-specified value (initial_carbon_io). During the reproductive phase, a carbon fraction a remobilization of a carbon fraction in the stem is allocated into reproductive structures such as panicles and grains. An equal process occurs for leaves, which simulates leaf senescence and reduces LAI. This happens when DVI exceeds the parameter that controls the stage of senescence (DVIsen = 0.4) (Equation (5)):

(5) $$\text{sen}\_\text{dvi}=\mu \;{\left(DVI-DVIsen\right)}^{\upsilon }$$

where μ and ν are allometric coefficients to calculate senescence.

Equal to the partition of carbon, the SLA is calculated as a DVI function as demonstrated in equation (6):

(6) $$SLA=\gamma \;{\left(DVI+0.06\right)}^{\delta }$$

where the coefficients δ and γ were obtained from allometric adjustments and the ratio of leaf dry mass and its carbon fraction ratio.

The green LAI is calculated using the SLA and the leaf carbon (Equation (7)):

(7) $$LAI=\frac{C\;leaf}{fc,leaf}\;\text{SLA}$$

where C leaf is the carbon pool in leaves and f_c,leaf_ is the carbon fraction in the dry leaves.

When the DVI hits 2, harvest is normally triggered, although it can be initialized sooner in certain circumstances (for example, excessive LAI values, low soil temperature, poor content of carbon in plants, and very sluggish crop development; see Ref. [21] for a more complete description). None of the simulations presented in this study initiated the early harvest procedure.

Crop height (h) is calculated using the Cstem pool as described in equation (8):

(8) $$h=k{\left(\frac{Cstem}{fc,stem}\right)}^{\lambda }$$

where k and λ are allometric parameters, and the f_c,stem_ is the fraction of carbon in dry stem including reserve.

### 2.2 Model configuration and spatial distribution of maize production

**Fig. 1:**
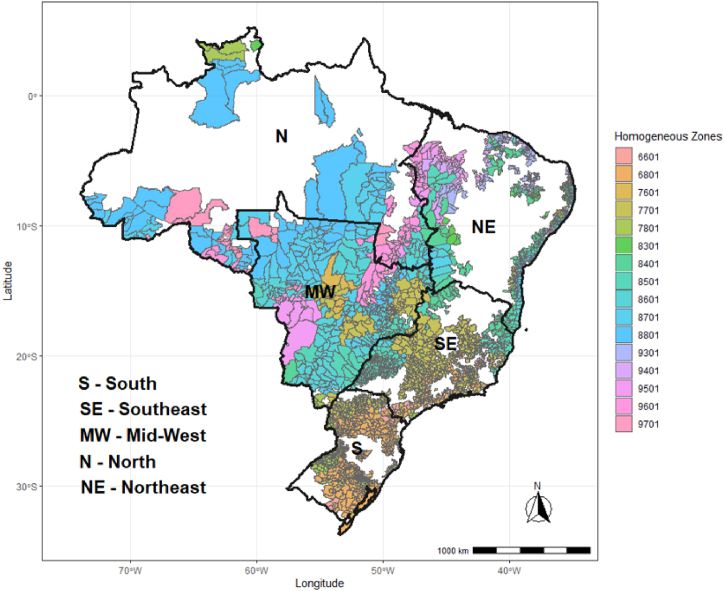
Brazilian territory and their respective climate homogeneous zone utilized in the simulations for predicting maize yield in off-season.

Due to the purposes of this study, we divided Brazil into 16 climate homogenous zones [25], which account for 92 % of Brazilian maize output during the off-season (Fig. 1). Simulations were carried out for one representative county of maize production in each CZ (Table 1). Hourly meteorological data spanning from 2003 to 2016 were obtained from the WATCH dataset, derived from ERA-Interim (WFDEI) re-analysis data(Weedon et al., 2018)[26]. Maize growth simulations were conducted in the most prevalent soil classes in each CZ (more than 10 %), as detailed in Table 1. JULES-crop was previously calibrated using a large diversity of cultivars in different sites across Brazil (Table 1). The calibration procedure was made for different JULES-crop outputs as leaf area index, crop height, soil moisture and leaf, stem and grain dry mass, being adjusted numerical constants related to the carbon allocation as described in equation (4), after a local sensitivity analysis detected these parameters as the most sensitivities. After, a leave-one-out cross-validation was made as a strategy to calibrate JULES-crop in different sites of Brazil, reaching high efficiency of modeling in main outputs as LAI (EF = 0.73), crop height (EF = 0.89) and grain dry mass (0.61). More details about methodology and results are described in Prudente Jr et al. [27]. To examine maize yield in different growing regions of Brazil, simulations were set up using the most common date of sown in each CZ (Table 1), which was collected from Cruz et al. [28] and Duarte and Sentelhas [29]. Simulations of harvest date used in the JULES-crop model, with consideration given to coincide to the physiological maturation of each cycle, indicated DVI value of 2.0 (Osborne et al., 2015; [21]).

**Table 1:** Counties description represented with each respective climate homogeneous zone in Brazil serves as a reference for developing maize yield forecasts using the outputs of the JULES-crop model and agrometeorological indicators.

CZ	Latitude	Longitude	Region	County	Sowing date	Cultivars^a^	Temperature and Rainfall (^o^C and mm) b	Plant Population	Row spacing (m)	Soil classification	(%) soil
7601	−22.037	−55.707	Midwest	Ponta Porã	1-Feb	DKB393	23.14, 432	65000.00	0.45	Rhodic Ferralsol	34.41
										Dystric Cambisol	16.32
6601	−23.938	−48.786	Southeast	Itapeva	1-Feb	DKB 363	21.27, 345	66000.00	0.45	Rhodic Ferralsol	46.18
										Ferric Acrisol	32.30
8801	−12.265	−58.004	Midwest	Brasnorte	1-Feb	DKB393	26.10, 604	65000.00	0.45	Ferric Acrisol	58.51
										Rhodic Ferralsol	13.66
										Ferralic Arenosol	11.00
8601	−12.342	−52.533	Midwest	Querência	1-Feb	DKB393	26.95,554	65000.00	0.45	Rhodic Ferralsol	52.38
										Ferralic Arenosol	13.30
8701	−12.705	−55.686	Midwest	Sorriso	1-Feb	DKB393	26.54,669	65000.00	0.45	Rhodic Ferralsol	41.04
										Ferric Acrisol	20.06
										Ferralic Arenosol	12.67
9501	−8.224	−46.842	North	Campos Lindos	1-Feb	DKB393	26.40,471	65000.00	0.45	Ferric Acrisol	21.13
										Rhodic Ferralsol	16.05
										Albic Plinthosol	15.91
8401	−9.543	−46.113	Northeast	Alto Parnaíba	1-Mar	AG1051	26.50,269	58823.00	0.85	Dystric Leptosol	33.94
										Ferralic Arenosol	33.05
										Rhodic Ferralsol	33.01
8501	−8.495	−46.533	Northeast	Balsas	1-Mar	AG1051	24.83,229	58823.00	0.85	Rhodic Ferralsol	45.81
										Albic Plinthosol	14.22
										Dystric Leptosol	12.24
										Ferric Acrisol	10.70
9401	−8.280	−45.841	Northeast	Tasso Fragosso	1-Mar	AG1051	26.86,267	58823.00	0.85	Dystric Leptosol	36.57
										Rhodic Ferralsol	34.33
										Albic Plinthosol	16.63
9301	−8.134	−45.486	Northeast	Ribeiro Gonçalves	1-Mar	AG1051	26.42,251	58823.00	0.85	Rhodic Ferralsol	60.16
										Dystric Leptosol	29.86
8301	−11.898	−45.411	Northeast	Barreiras	1-Apr	AG1051	26.76,167	58823.00	0.85	Rhodic Ferralsol	80.30
7701	−17.699	−51.015	Midwest	Rio Verde	1-Feb	DKB393	24.70,414	65000.00	0.45	Rhodic Ferralsol	40.84
										Ferralic Arenosol	22.50
7801	−24.594	−52.804	South	Campina da Lagoa	1-Feb	Pionner 3230	20.87,437	67000.00	0.75	Ferric Acrisol	59.03
6801	−24.728	−53.242	South	Corbelia	1-Feb	Pionner 3230	20.68,492	67000.00	0.75	Rhodic Ferralsol	41.91
										Dystric Leptosol	17.03
										Dystric Cambisol	15.09
										Rhodic Nitisol	10.82
9701	−13.536	−60.603	North	Cabixi	1-Apr	DKB393	24.39,115	65000.00	0.45	Rhodic Ferralsol	38.80
										Albic Plinthosol	31.60
9601	−13.108	−61.599	North	Pimenteiras do Oeste	1-Apr	DKB393	25.01,127	65000.00	0.45	Rhodic Ferralsol	50.15
										Albic Plinthosol	34.11

a Parameters related to specific cultivar were based on Prudente et al. (2022).

b Total annual precipitation and average air temperature observed during the off-season maize in each CZ during the period 2003–2016.

### 2.3 Agrometeorological indicators and JULES-crop outputs

Due to developing a model to predict maize yield, simulations related to potential (Yp) and water-limited yields (Yw) were made and generated agrometeorological variables for each CZ, divided by 10 days of the cycle from sown to harvest date (Table 2). Based on Pagani et al. [15], we employed two soil water outputs related to hydric balance from JULES-crop during the cycle for the water-limited simulations: the soil water content (SWC) in the root zone and the water stress factor (FSMC), which ranges from 0 in extreme drought stress to 1 when there is no drought stress. The SWC parameter acquired from the simulation was calculated as the difference between soil moisture and residual soil moisture; however, we used 0 for soil moisture residual because there was no significance in the values in each location. For both soil properties, we adopted the average of each 10 cm depth taking into account the effective root zone of maize (60 cm).

**Table 2:** List of model outputs of JULES-crop and agrometeorological indicators selected each 10-day period and utilized to predict yield.

Indicator name	Unit	Production level	Description
Model outputs			
LDM	Mg ha^−1^	P^a^, WL^b^	Leaf dry mass
SDM	Mg ha^−1^	P, WL	Stalk dry mass
GDM	Mg ha^−1^	P, WL	Grain dry mass
CH	M	P,WL	Crop height
LAI	m^2^ m^−2^	P,WL	Leaf area index
FSMC	0–1	WL	Integer indicating weighting of soil layers in water stress factor
SWC	m^3^ m^−3^	WL	Soil water content in the rooted zone
Agrometeorological indicator			
TMED	^o^ C	P,WL	Average daily medium temperature from sowing to the date
RAIN	Mm	WL	Accumulated rainfall from sowing to the date
DIFF_RAD	MJ m^−2^ day	P,WL	Average diffuse radiation from sowing to the date

a Potential yield.

b Water-limited potential yield.

The analysis also includes outputs of JULES-crop that are straight connected to maize yield, such as leaf dry mass (LDM), stalk area index (SDM), grain dry mass (GDM), leaf area index (LAI), and crop height (CH). The agrometeorological indicators chosen were diffuse incoming radiation (DIFF_RAD), accumulated precipitation (RAIN), and average air temperature (TMED) from sown to every 10-day period until harvest (120 days), as provided by WFDEI, except diffuse radiation, which was calculated using Weiss and Norman's [30] method.

Outputs of JULES-crop and agrometeorological variables were weighted based on off-season maize cultivated area in each CZ, using data provided by official statistical data from IBGE (www.ibge.gov.br) during the years 2003–2016. The first step was to calculate a weighted average using the off-season maize cultivated area in each CZ's producer counties. The second step was to calculate the weighted average based on each CZ's farmed area participation in Brazil, resulting in a national scale. For the maize simulation results generated by JULES-crop, a weighted average was derived to approximate the soil percentage (Table 1) in each CZ.

### 2.4 Statistical analysis

Table 2 outlines the outputs of JULES-crop and agrometeorological indicators used as independent variables for multiple linear regressions covering the time series from 2003 to 2016 to predict grain yield at a national scale. Each 10-day period from sown date to the harvest date, as detailed in Table 1, was considered. To eliminate the influence of significant technological trends such as advancements in management practices and cultivars during the period, a detrending procedure following an approach described by Pagani et al. [15] was applied.

To approach the diversity of farmers practices in water management on maize yield, the regressors were divided into three groups.

1 Yp for JULES-crop outputs under potential condition
2 Yw for JULES-crop outputs under non irrigated (water-limited) condition
3 Agrometeorolgical indicators

Due the necessity to identify the most suitable regressors explaining yield inter-annual variability in each 10-day period from the sown a stepwise analysis was conducted.

To mitigate overfitting and collinearity issues in each regressor, The Variance Inflation Factor (VIF, 1 to +∞, optimum = 1) was computed. This calculation is described by equation (9):

(9) $$\text{VIF}=\frac{1}{1-R_{i}^{2}}$$

Where the variance proportion is R^2^_i_ as the *i*th independent variable regarding the other independent variable presented in the model. Another statistical index analyzed was the absence or presence of autocorrelation among residuals, for this implemented the Durbin and Watson test ([31], Equation (10)):

(10) $$\text{DW}=\frac{\sum_{t=2}^{n}{\left(e_{t}-e_{t-1}\right)}^{2}}{\sum_{t=1}^{n}e_{t}^{2}}$$

where e_t_ is the difference between observed and predicted yield in the *t*th year of the time series; n is the number of years. The evaluation of the absence of autocorrelation among residuals was based on Savin and White (1977). The Durbin-Watson test result is considered.

• Accepted in case the calculated statistic is higher than the upper critical value (dU),
• rejected in case the calculated statistic is smaller than the lower critical value (dL),
• inconclusive in case the calculated statistic falls between dL and dU.

The *t*-test was employed to assess the significance (p-value) of each indicator in the regression model and its effects during the cycle. For the evaluation of the regression model performance, a leave-one-out cross-validation method [32] was utilized. This involved removing one year of anticipated output and comparing it to historical yields. Several goodness-of-fit measures were selected, including the Coefficient of determination (R^2^); Index of agreement (d) (Wilmott et al.,2012) [33]; Nash Stucliffe efficiency index (EF) (Nash-Sutcliffe,1970) and the Standard deviation error in prediction (SDEP, Mg ha^−1^) as described by Stock and Watson [34]. These measures were chosen based on prior studies by Marin et al. [35], Pagani et al. [15] and Wallach et al. [32].

## 3 Results

### 3.1 Statistical model selection

In each forecasting window, the best regression models were selected using a stepwise analysis of JULES crop outputs and agrometeorological indicators (Table 3). All regressor combinations had a significance level of less than 0.05 for the 80–120 days of the 10-day period (Table 3), indicating that the regressor models performed best during the maize reproductive stage, from the start of grain filling to maturity. The DW test was inconclusive in determining whether to accept or reject the among residuals correlation in most 10-day intervals; nevertheless, the null hypothesis of the DW test was accepted at 10, 50, and 120 days after sowing (Table 3). In inconclusive circumstances, the null hypothesis could not be refuted. The VIF values in each regressor model are never greater than 5.0 (Table 3), indicating the lack of multicollinearity. In terms of regressor model performance, the EF increased as the model approached maturity, peaking between the eightieth and last 10-day period (EF = 0.65 and EF = 0.77, respectively). During the vegetative phase, the lowest EF values were found during in the first ten days after sowing (EF = 0.43) and on the fifty-first day of the cycle (EF = 0.46).

**Table 3:** Each forecasting window and its statistical performance indexes are based on regressor models developed for off-season maize in Brazil using outputs of JULES-crop and agrometeorological indicators.

Statistical index	Days after sowing											
	10	20	30	40	50	60	70	80	90	100	110	120
R^2^	0.43	0.53	0.48	0.52	0.46	0.55	0.60	0.71	0.65	0.65	0.72	0.77
d	0.77	0.82	0.80	0.81	0.79	0.84	0.84	0.91	0.89	0.89	0.92	0.93
EF	0.43	0.53	0.48	0.52	0.46	0.55	0.60	0.71	0.65	0.65	0.72	0.77
VIF	1.75	2.13	1.92	2.08	1.85	2.22	2.50	3.45	2.86	2.86	3.57	4.35
DW	1.61	1.76	1.34	1.28	1.53	1.24	1.31	1.47	1.24	1.45	1.73	1.83
p-value	0.116	0.110	0.081	0.126	0.095	0.097	0.128	0.044	0.033	0.036	0.037	0.001

### 3.2 Selected indicators for regressors models

**Fig. 2:**
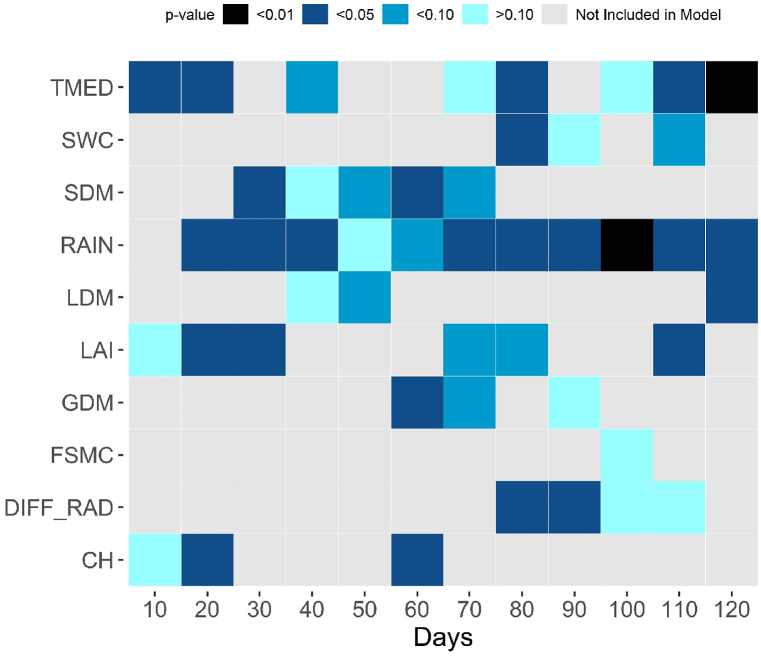
Heatmap representing each forecasting window selected based on outputs of JULES-crop in water-limited (Yw) conditions and agrometeorological indicators for off-season maize across Brazil. Notably, none of the outputs of JULES-crop in potential condition (Yp), was detected by the stepwise regression method.

The stepwise analysis revealed the elements (independent variables) impacting grain production (dependent variable) over each 10-day period, in comparison to official yields in Brazil (Fig. 2). A heatmap was created to display for each regression model the main significant indicators using agrometeorological indicators and maize outputs simulated for water-limited conditions with improved statistical performance. RAIN (Fig. 2) was the most relevant element detected during the maize cycle, which was selected by the stepwise analysis in all regression model. The exception was at the first 10 days, this indicator was not detected. Another essential agrometeorological indicator was TMED, being more significant on the 120th day of the maize maturation. Only agrometeorological indicators explained 60 % of the variability in maize yield on days 80, 90, 110, and 120 (reproductive and maturity stages). LAI and SDM were the most often designated variables obtained by JULES-crop outputs throughout the cycle, and they were particularly significant during the vegetative stage. The regression models only defined the SWC 80 days after planting, which is a period of low rainfall in most major CZ maize producers during the off-season in the Brazilian Midwest and Southeast regions, respectively.

### 3.3 Yield forecast evaluation

**Fig. 3:**
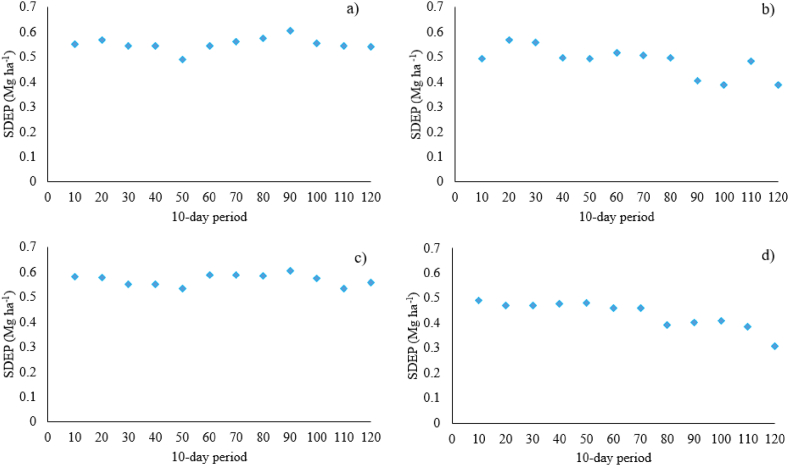
Standard deviation error in prediction (SDEP) of different forecasted yield for off-season maize in Brazil based on a) JULES-crop outputs in water-limited condition; b) agrometeorological indicators; c) JULES-crop outputs in potential conditions; d) JULES-crop in water-limited conditions + agrometeorological indicators.

**Fig. 4:**
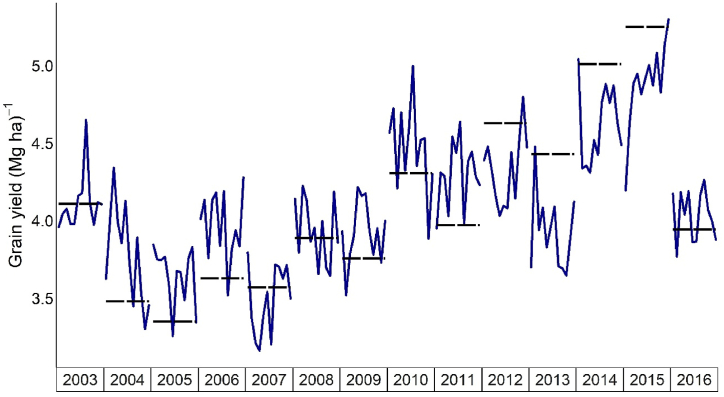
Official off-season grain maize yield (dotted lines) compared with yield simulated during the maize cycle for each 10-day time window (solid line).

In view of to analyze the effectiveness of using outputs simulated by JULES-crop in water-limited and potential conditions, was necessary to calculate the SDEP of each forecasting window. It was discovered that none of the 10-day periods favored outputs related to JULES-crop in potential condition by the stepwise regression, owing to its poor statistical performance indexes during the cycle (Table 3, Fig. 3c). Overall, agrometeorological indicators proved effective in explaining the variability presented in different years that justify maize production in Brazil, primarily during the reproductive phase, with SDEP ranging from 0.5 to 0.38 Mg ha^−1^ (80–120 days, respectively) in this phenological stage (Fig. 3b). Nevertheless, it was shown that utilizing outputs of JULES-crop and agrometeorological indicators jointly in regression models during the reproductive period (80–120 days) reduced the SDEP by 0.4 to 0.31 Mg ha^−1^ (Fig. 3d). Improvements of JULES-crop water limiting outputs and agrometeorological indicators were detected during the vegetative stage (Fig. 3d), with SDEP varying from 0.49 to 0.46 Mg ha^−1^, whereas others had SDEP ranging from 0.59 to 0.49 Mg ha^−1^ until the 70th day. Moreover, only outputs of JULES-crop in water-limited settings justified the variability of grain yield, primarily in the vegetative stage at 30 and 50 days (SDEP = 0.54 and 0.49 Mg ha^−1^, respectively), when leaves (LDM and LAI) and SDM outputs predominated (Figs. 2 and 3a). Fig. 4 depicts the simulated off-season maize yield, which has the highest statistical indexes of performance when compared to official data. Every year, the regression models achieved the official yield in any 10-day period, even the years that presented the highest official yields (2014 and 2015). Furthermore, it was indeed possible to anticipate results close to the official data from the 80th day of the cycle throughout the years 2003–2016, with days 80, 110, and 120 being the most similar to the official yield data for off-season maize for Brazilian territory (Figs. 2 and 4).

### 3.4 Potential application of the maize forecast model

**Fig. 5:**
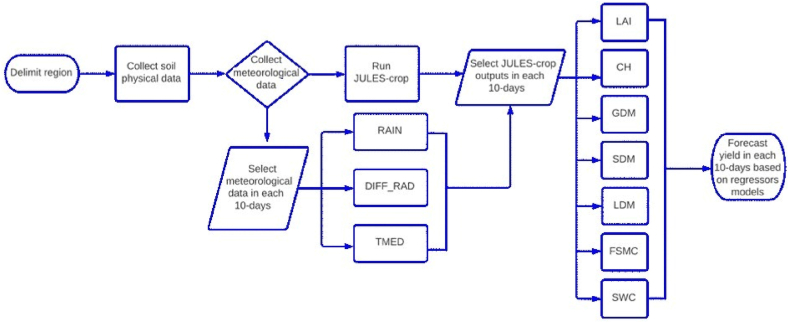
Workflow of the procedures to forecast maize yield using JULES-crop outputs and agrometeorological indicators.

In view of the potential application of the forecast model presented in this study, a workflow was made with the aim of simplifying the procedure (Fig. 5). In Brazil, the main meteorological data source available to the public is the INMET (National Meteorological Institute). INMET updates meteorological data everyday with hourly data of air temperature (^o^C), incident solar radiation (MJ m^−2^ h^−1^), air pressure (kPa), precipitation (mm), wind velocity (m s^−1^) and air humidity (%) in 567 automatic weather stations distributed in different regions of Brazil. Two adaptations are recommended using this data information to estimate the diffuse radiation and the downward longwave radiation. In the first case, we recommended estimating the diffuse radiation using the Weiss and Norman [30] method and for the second case, due to the fact of demand of JULES-crop run simulations with short and longwave radiation, we recommended the global radiation as the downward shortwave radiation and the estimation of downward longwave radiation is estimated using Prata [36].

## 4 Discussion

The forecast yield model for off-season maize established in this study identified TEMP and RAIN as the primary drivers of inter-annual variability in maize production in Brazil (Fig. 2). Temperature affects the accumulation of maize biomass by influencing the length of the growing season and the average daily growth rate [37,38]. Furthermore, temperatures beyond the optimum level (32^o^C for maize) might result plant tissue damage and pollination failures as metabolic activity increases (Johkan et al., 2011; [39]). The condition of temperature above the optimum level for maize was found in many CZs of our study such as in the Northeast and Midwest region. Rainfall is responsible to attend crop water demands, and maize is highly sensitive to drought, particularly during the reproductive stage, which affects the efficiency of photosynthesis due to stomatal closure and leaf wilting [40,41]. In contrast, we find that two water availability related output (SWC and RAIN, Fig. 2) had the most relevance during the period of grain-filling (around 80th day) being the stage with highest sensitivity during the cycle [42]. The diffuse radiation was taken into account in this study and showed high significance during the grain-filling period (Fig. 2) as observed by Liu et al. [43], which detailed an increment of 5.9 % in grain production under high solar radiation incidence during grain-filling. Some outputs of JULES-crop such as FSMC and LDM were selected with lower frequency during the cycle in comparison to the others (Fig. 2). The FSMC output is related to hydric stress, and it is directly related to the SWC output, being the second calibrated by Prudente Jr et al. [27] (EF:0.44), the accuracy and feasibility of SWC could be the reason to be less selected. LDM output is related to the LAI output and the second presented more modeling efficiency in the calibration procedure developed by Prudente Jr et al. [27] (EF LAI: 0.73; EF LDM:0.39). High accuracy of JULES-crop to simulate LAI was also observed by Williams et al. [21] when developed a new parameterization of JULES-crop for maize in Nebraska-USA.

JULES-crop outputs and agrometeorological indices integrated were profitable in explaining the yield variability in all considered forecast windows. A system to predict maize yield in Northeast Brazil was developed by Martins et al. [4] using crop model and agrometeorological indices based on the integretation between weather data and Aqua-crop model, reaching consistent forecasts at least 30 days before harvest, in order to that found in our study. During the grain-filling period a high performance was detected (R^2^ = 0.71) in this study, also it was observed by those authors with similar precision (R^2^ = 0.74). An equal situation was also observed by Bussay et al. [44] who identified a high precision in predicting maize yield in Hungary during the grain-filling stages. In this study, the authors used the outputs of the process-based model WOFOST to forecast maize yield and observed the highest R^2^ and the lowest RMSE at the grain-filling stage (R^2^ = 0.85 and RMSE = 0.43 Mg ha^−1^). The RMSE observed in Bussay et al. [44] is similar than observed in this study (RMSE = 0.41 Mg ha^−1^) despite the R^2^ is lower in comparison of this study (R^2^ = 0.71), this could be explained by the climate variability in Hungary territory due the large climate diversity across Brazil, being more challenger to develop forecast models with high precision in largest countries. Soler et al. [17] used the CERES-maize model to predict off-season maize and forecasted maize yield with reasonable performance around 45 days prior the harvest in Southern Brazil. For other crops such as millet [45] and peanut [46], yield forecasts approaches also verified an improvement of prediction after the vegetative stage, being justified by the canopy establishment and the grain development, which is the main focus in crop forecasting models. This also can explain the inconclusive results of DW test (Table 3) presented in this study at the reproductive stage of the cycle. DW test inconclusive was also observed by Pagani et al. [15] in a yield forecast model of sugarcane, being explained by the low value of LAI and canopy height during the reproductive stage.

None of the JULES-crop potential growth outputs had a significant impact on the forecasting performance at any crop stage. Thereby, they were not selected by the stepwise analysis employed in the proposed forecast model. Pagani et al [15] developed a forecast yield system for sugarcane to be used in the State of São Paulo based on agrometeorological indicators and outputs of DSSAT/Canegro, and in none of the forecast windows examined, the potential associated variables were chosen using the stepwise analysis. The authors also discovered that in water-limited situations, a combination of agrometeorological indicators and crop model output performed best for predicting sugarcane yield. One probable justificative to deny the crop outputs simulated under potential conditions is Brazilian management, which is known for not irrigating maize during the off-season, during a period when precipitation reduces near the reproductive stage in the Brazilian center-south, resulting in a production gap of 3.2 Mg ha-1 [6]. Another possible reason is the characteristic in the major of a process-based model to overestimating crop yield in potential conditions, presenting some difficulties to simulate the effects of high temperatures mainly during the flowering phase, the same situation was related by Bussay et al [44] that reached high performance using WOFOST model using water limited output (LAI and Above ground biomass). Besides this possibility, it is necessary to take into account the climate diversity across Brazilian territory and the farmers’ features to improve their agriculture management, especially in off-season maize that requires water in the moment when the rainfall decreases, and the air temperature increases in different ranges. Thus, the outputs related to the water-limited condition were selected by the stepwise regression.

Similar to the strategy adopted in our study, Coelho and Costa [47] and Bergamashi et al. [48] used a large-scale model (GLAM) for forecasting maize production in Brazil, with a high precision (R^2^ = 0.77) for predicting maize yield in the maturation forecast window, near to the value that we found here. Given this study developed a yield forecast for maize that can be nationality used, outputs provided by large scale model cooperated to generating predictions with similar precision and during the same stage of regional scale studies. In view of the proposal to use the prediction model developed in this study to forecast maize production in the next years, the workflow produced (Fig. 5) serves as a guide for operational decisions in front of the possibility to be applied with different meteorological data source, only with some adjustments in the units and the in estimation of longwave and diffuse radiation. Some aspects could be improved in the forecast model as the analysis in grid (0.25^o^ x 0.25^o^) as approached by Pagani et al [15] and the utilization of another agrometeorological indicators such as evapotranspiration and wind velocity; however, with the indicators pointed we developed an operationally viable forecast model with precision and accuracy to predict maize yield in large scale with 40 days in advance.

## 5 Conclusion

The presented study provided a forecast model to predict maize yield in off-season for Brazilian territory based on outputs of JULES crop and agrometeorological indices. A stepwise analysis identified components of a regression model presented in each forecast window, with rainfall and temperature accounting for 60 % of the off-season maize inter-annual variability in Brazil between 2003 and 2016. Furthermore, outputs of JULES-crop represented an improvement of the ability to predict during the reproductive stage, accounting for 77 % of yield variance in the maturation, even as outputs related to stem and leaf dry mass, which decreased error when combined with the agrometeorological indicator during the vegetative period. The prediction model developed in this study accurately forecasted with high precision maize yield around 40 days before harvest (p-value <0.05) from the grain-filling period. Finally, this study demonstrated that JULES-crop might contribute to a large-scale national forecast of one of Brazilian most important agricultural commodities.

## Funding information

The funding sources for this research include the Research Foundation of the State of São Paulo (10.13039/501100001807FAPESP 2017/20925-0, 2021/00720-0), Brazilian Research Council (10.13039/501100003593CNPq grants 425174/2018-2 and 300916/2018-3), and the Coordination for the Higher Education Improvement – Brazil (10.13039/501100002322CAPES). Additionally, the contributors and its study received support from the Newton Fund through the 10.13039/501100000847Met Office Climate Science for Service Partnership Brazil (CSSP Brazil).

## Data availability statement

Data will be made available on request.

## CRediT authorship contribution statement

**Amauri Cassio Prudente Junior:** Writing – review & editing, Writing – original draft, Visualization, Validation, Supervision, Software, Resources, Project administration, Methodology, Investigation, Formal analysis, Data curation, Conceptualization. **Murilo S Vianna:** Writing – review & editing, Writing – original draft, Validation, Supervision, Software, Methodology, Formal analysis, Data curation, Conceptualization. **Karina Willians:** Writing – review & editing, Writing – original draft, Visualization, Validation, Software, Funding acquisition, Formal analysis, Data curation, Conceptualization. **Marcelo V Galdos:** Writing – review & editing, Writing – original draft, Software, Resources, Methodology, Funding acquisition, Formal analysis, Data curation, Conceptualization. **Fabio R. Marin:** Writing – review & editing, Writing – original draft, Visualization, Validation, Supervision, Software, Resources, Project administration, Methodology, Investigation, Funding acquisition, Formal analysis, Data curation, Conceptualization.

## Declaration of competing interest

The authors declare that they have no known competing financial interests or personal relationships that could have appeared to influence the work reported in this paper.
